# Performance and acute procedural outcomes of the EnSite Precision™ cardiac mapping system for electrophysiology mapping and ablation procedures: results from the EnSite Precision™ observational study

**DOI:** 10.1007/s10840-022-01239-4

**Published:** 2022-05-10

**Authors:** Jonathan C. Hsu, Douglas Darden, Benedict M. Glover, B. Judson Colley, Christian Steinberg, Bernard Thibault, Coty Jewell, Michael Bernard, Paul B. Tabereaux, Usman Siddiqui, Jingyun Li, Eric E. Horvath, Daniel Cooper, David Lin

**Affiliations:** 1 University of California San Diego, 4952 Medical Center Dr, ACTRI Bldg, 3rd Floor, Room 3E-313, MC7411, La Jolla, CA 92037 USA; 2 University of Toronto, Toronto, Canada; 3 Jackson Heart Clinic, Jackson, MS USA; 4 Institut Universitaire de Cardiologie Et Pneumologie de Québec (IUCPQ-UL), Laval University, Quebec, Canada; 5 Montreal Heart Institute, Montreal, Canada; 6 Oklahoma Heart Hospital South, Oklahoma City, OK USA; 7 Ochsner Medical Center, Jefferson, LA USA; 8 Heart Center Research, LLC., Huntsville, AL USA; 9 Florida Electrophysiology, Atlantis, FL USA; 10 Abbott Laboratories, Zephyrhills, FL USA; 11 Washington University School of Medicine, St. Louis, MO USA; 12 Hospital of the University of Pennsylvania, Philadelphia, PA USA

**Keywords:** Electroanatomical mapping, Catheter ablation, Image processing, Ensite mapping system

## Abstract

**Background:**

The EnSite Precision™ cardiac mapping system (Abbott) is a catheter navigation and mapping system capable of displaying the three-dimensional (3D) position of conventional and sensor-enabled electrophysiology catheters, as well as displaying cardiac electrical activity as waveform traces and dynamic 3D maps of cardiac chambers. The EnSite Precision™ Observational Study (NCT-03260244) was designed to quantify and characterize the use of the EnSite Precision™ cardiac mapping system for mapping and ablation of cardiac arrhythmias in a real-world environment and evaluate procedural outcomes.

**Methods:**

A total of 1065 patients were enrolled at 38 centers in the USA and Canada between 2017 and 2018 and were followed for 12 months post procedure for arrhythmia recurrence, medication use, and quality-of-life changes. Eligible subjects were adults undergoing a cardiac electrophysiology mapping and radiofrequency ablation procedure using the EnSite Precision™ System.

**Results:**

A final cohort of 925 patients (64.3 years of age, 30.2% female) were analyzed. The primary procedural indication was atrial flutter in 48.1% (445/925), atrial fibrillation in 46.5% (430/925), and other arrhythmias in 5% (50/925). Electroanatomic mapping was performed in 81.5% (754/925) of patients. Mapping was stable throughout 79.8% (738/925) of procedures with initial mapping time of 8.6 min (IQR 4.7–15.0). Average mapping efficiency created with AutoMap or TurboMap was 164.9 ± 365.7 used points per minute. Median number of mapping points collected and used was 1752.5 and 811.0, respectively. Only 335/925 (36.2%) required editing and 66.0% (221/335) of these patients required editing of less than 10 points. Fluoroscopy was utilized in most cases (*n* = 811/925, 87.4%) with fluoroscopy time of 11.0 min (IQR 6.0–18.0). Overall median procedure time was 101.0 min (IQR 59.0–152.0). Acute procedural success was high for both atrial fibrillation (*n* = 422/430, 98.1%) and atrial flutter (*n* = 434/445, 97.5%).

**Conclusion:**

In a real-world study analysis, use of the EnSite Precision™ mapping system was associated with high procedural stability, short mapping times, high point density requiring infrequent editing, low fluoroscopy time, and high prevalence of acute procedural success.

**Supplementary Information:**

The online version contains supplementary material available at 10.1007/s10840-022-01239-4.

## Introduction

Electroanatomic mapping (EAM) has become an essential tool for effective ablation of cardiac arrhythmias with continued advancements in three-dimensional (3D) catheter tracking and high-resolution visualization to account for the increased case complexity and duration [1, 2]. Available since 2016, the EnSite Precision™ cardiac mapping system (Abbott, St. Paul, Min) uses a hybrid impedance and magnetic field technology that displays the 3D position of conventional and sensor-enabled electrophysiology catheters, as well as generating high-density automated 3D electroanatomic maps that aim to improve success in complex ablation procedures [3, 4].

The EnSite Precision™ Observational Study was designed to quantify and characterize the use of the EnSite Precision™ cardiac mapping system for mapping and ablation of cardiac arrhythmias in a real-world environment and evaluate procedural and subsequent clinical outcomes. In this report, we describe the performance of the system including mapping stability, mapping times, points collected, fluoroscopy times, and acute procedural success.

## Methods

### Study design

The EnSite Precision™ Observational Study (NCT-03260244) was designed to quantify and characterize the use of the EnSite Precision™ cardiac mapping system for mapping and ablation of cardiac arrhythmias in a real-world environment and evaluate procedural and subsequent clinical outcomes. There was no randomization or blinding in this study.

The study was designed and sponsored by Abbott Laboratories and approved by the appropriate Institutional Review Board or Ethics Committee at each site. Data monitoring, collection, and primary data analysis were performed by the sponsor in partnership with the publication committee. This clinical study was conducted in accordance with Abbott Standard Operating Procedures, ethical principles based on the Declaration of Helsinki, Good Clinical Practice, ISO 14155, and FDA 21 CFR 50, 54, 56, and 812.

### Study population

Eligible subjects were adults undergoing a cardiac electrophysiology mapping and radiofrequency (RF) ablation procedure using the EnSite Precision™ System. Subject enrollment in the EnSite Precision Observational Study began on September 12, 2017. The last subject was enrolled on December 6, 2018. The study enrolled 1065 subjects at 38 clinical sites in the USA and Canada. A subject was considered enrolled in the clinical study from the moment the subject provided a written informed consent. Subjects were followed for 12-months post procedure for arrhythmia recurrence, medication use, and quality-of-life changes. The last follow-up visit was completed on January 17, 2020.

Patients with atrioventricular nodal reentrant tachycardia (AVNRT) or atrioventricular reentrant tachycardia (AVRT) as the only presenting rhythm and patients with planned cryoablation procedure were excluded. A full list of inclusion and exclusion criteria is included in Supplemental Table 1.

### Study procedures

Subjects underwent a cardiac mapping and RF ablation procedure using the EnSite Precision™ cardiac mapping system per standard practice of the operating physician. Subjects were prepared according to the standard ablation procedures and standard practice of the center. All devices had proper regulatory clearance and were used according to their IFU, including anticoagulation and activated clotting time therapeutic requirements for multi-electrode catheters. Procedure data collection included overall procedure (first catheter in, to last catheter out), fluoroscopy, and mapping times. EnSite NavX Surface Electrode (NavX patch) placement and associated skin preparation were recorded. Documented mapping characteristics included times to create and edit the initial map; number of mapping points collected, used, and edited; and EnSite™ Automap and AutoMark module software settings used. Editing included reannotating or deleting previously collected points. Time spent “shaving” was not specifically captured. The number of gaps in lesions identified that required further ablation (touch-ups) was also recorded. Operators were asked to note whether the mapping system was stable throughout the procedure (based on the opinion of the operator) and any factors affecting system stability. Mapping efficiency for a given map was characterized as the number of used points divided by the mapping time in minutes, resulting in used points per minute.

### Study outcomes

Acute success was defined by the operator based upon their standard pre-defined endpoints for each type of procedure. Adverse events were not collected during this clinical study. Long-term follow-up up to 12-months will be reported in a separate analysis. Any complaints were managed via the sponsor’s standard post-market surveillance process.

### Statistical analysis

Continuous variables are summarized with number of observations, mean, standard deviation, min and max values, or median and interquartile range (IQR). Categorical variables are summarized with patient counts and percentages. All data available among the analysis population was used. Missing data was not imputed. No formal sample size calculation was performed. All analyses were performed using SAS software version 9.4 (SAS Institute Inc, Cary, NC, USA). The *p*-values presented are 2-sided, and *p* < 0.05 (not adjusted for multiplicity) was considered statistically significant.

## Results

### Enrollment and analysis population

Of the 1065 enrolled subjects, 1053 met all inclusion/exclusion criteria. Of these, 69 were excluded due to a primary indication of persistent AF in the USA that was treated off-label. A total of 45 subjects withdrew prior to the procedure and an additional 14 subjects did not have an eligible procedure (no RF ablation performed).

**Fig. 1:**
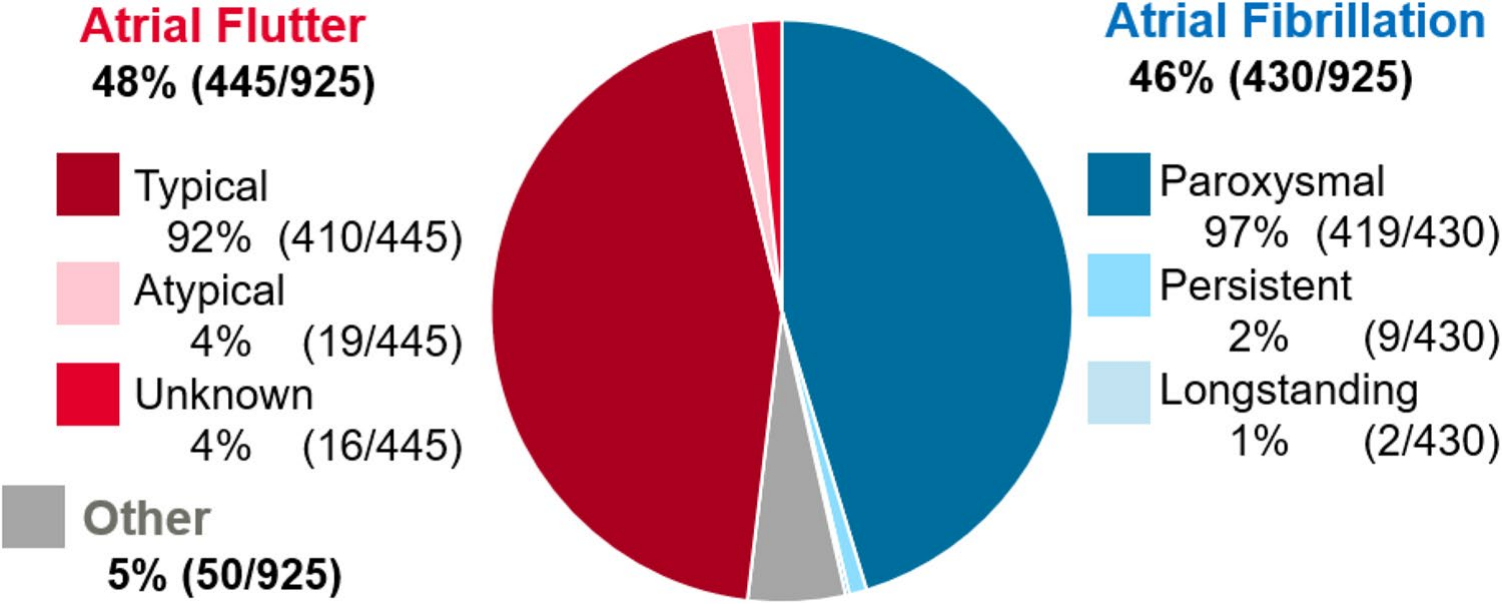
Primary indication for ablation procedure

The final cohort of 925 subjects stratified by primary indication for ablation include the following: AF (primary indication [PI]-AF, 46.5%, 430/925), AFL (PI-AFL, 48.1%, 445/925), or Other (PI-O, 5.4%, 50/925), as demonstrated in Fig. 1. The PI-O cohort included 18 supraventricular tachycardia (SVT), 11 atrial tachycardia, 9 premature ventricular contraction (PVC), 6 ventricular tachycardia, and 6 Wolff-Parkinson-White Syndrome patients. Additional lesions were common in the PI-AF cohort, including cavotriscupid isthmus (CTI) ablation (34.7%), lines excluding CTI (8.8%), complex fractionated atrial electrograms (4.0%), and rotor ablation (2.3%), as shown in Supplemental Table 2.

Baseline characteristics of the cohort are summarized in Table 1. The mean age was 64.3 ± 11.6 years, 646 (69.8%) were male, and mean body mass index was 30.9 ± 7.4 kg/m^2^. The majority (84.2%, 779/925) did not have an implantable cardiac device at the time of procedure. Mean left ventricular ejection fraction was 54.2 ± 11.9. The most prevalent cardiovascular diseases included history of hypertension (62.8%, 581/925), valvular heart disease (27.9%, 258/925), coronary artery disease (25.1%, 232/925), and history of diabetes (22.4%, 207/925). Of the 430 PI-AF subjects, 102 (23.7%) had a prior ablation for AF. Only 40 (9.0%) of 445 PI-AFL subjects had a prior ablation for AFL.

**Table 1:** Baseline characteristics stratified by primary indication for treatment

	AF	AFL	Other	All
	*N* = 430	*N* = 445	*N* = 50	*N* = 925
Demographics and baseline assessments				
Age (years)				
Mean ± SD (*n*)	63.5 ± 10.6 (430)	66.0 ± 11.5 (445)	56.2 ± 15.6 (50)	64.3 ± 11.6 (925)
(Min, Max)	(27.0, 86.0)	(25.0, 88.0)	(20.0, 80.0)	(20.0, 88.0)
Gender				
Female	33.7% (145/430)	24.7% (110/445)	48.0% (24/50)	30.2% (279/925)
Male	66.3% (285/430)	75.3% (335/445)	52.0% (26/50)	69.8% (646/925)
Height (in.)				
Mean ± SD (*n*)	68.6 ± 4.1 (430)	68.7 ± 3.8 (445)	66.7 ± 4.4 (50)	68.6 ± 4.0 (925)
(Min, Max)	(56.0, 81.1)	(55.0, 79.9)	(59.8, 78.0)	(55.0, 81.1)
Weight (lb)				
Mean ± SD (*n*)	210.6 ± 53.8 (430)	207.3 ± 50.9 (444)	175.5 ± 43.8 (50)	207.1 ± 52.4 (924)
(Min, Max)	(57.3, 657.0)	(105.6, 401.9)	(105.8, 286.6)	(57.3, 657.0)
Body mass index (kg/m^2^)				
Mean ± SD (*n*)	31.4 ± 7.8 (430)	30.8 ± 7.0 (444)	27.6 ± 5.7 (50)	30.9 ± 7.4 (924)
(Min, Max)	(9.3, 124.1)	(18.6, 60.9)	(14.9, 42.7)	(9.3, 124.1)
Implanted cardiac device				
None	81.4% (350/430)	86.7% (386/445)	86.0% (43/50)	84.2% (779/925)
Pacemaker	6.7% (29/430)	5.4% (24/445)	8.0% (4/50)	6.2% (57/925)
Implantable cardiac monitor	8.4% (36/430)	2.5% (11/445)	2.0% (1/50)	5.2% (48/925)
ICD	2.6% (11/430)	3.6% (16/445)	2.0% (1/50)	3.0% (28/925)
CRT-P	0.2% (1/430)	0.4% (2/445)	0.0% (0/50)	0.3% (3/925)
CRT-D	0.0% (0/430)	0.9% (4/445)	2.0% (1/50)	0.5% (5/925)
Other	0.7% (3/430)	0.4% (2/445)	0.0% (0/50)	0.5% (5/925)
LVEF (%)				
Mean ± SD (*n*)	56.5 ± 10.5 (323)	52.4 ± 12.5 (353)	51.3 ± 14.2 (35)	54.2 ± 11.9 (711)
(Min, Max)	(5.0, 82.0)	(15.0, 76.0)	(20.0, 80.0)	(5.0, 82.0)
NYHA classification				
I	4.2% (18/430)	3.1% (14/445)	2.0% (1/50)	3.6% (33/925)
II	5.8% (25/430)	5.4% (24/445)	12.0% (6/50)	5.9% (55/925)
III	1.9% (8/430)	2.2% (10/445)	2.0% (1/50)	2.1% (19/925)
IV	0.0% (0/430)	0.2% (1/445)	0.0% (0/50)	0.1% (1/925)
Not evaluated	88.1% (379/430)	89.0% (396/445)	84.0% (42/50)	88.3% (817/925)
Cardiovascular disease				
Coronary artery disease	25.6% (110/430)	25.6% (114/445)	16.0% (8/50)	25.1% (232/925)
Myocardial infarction	7.4% (32/430)	9.4% (42/445)	6.0% (3/50)	8.3% (77/925)
Previous CABG	6.5% (28/430)	11.0% (49/445)	4.0% (2/50)	8.5% (79/925)
Percutaneous coronary intervention/stent/atherectomy	11.6% (50/430)	12.1% (54/445)	8.0% (4/50)	11.7% (108/925)
Cardiomyopathy	13.5% (58/430)	17.8% (79/445)	20.0% (10/50)	15.9% (147/925)
Valvular heart disease	27.7% (119/430)	28.8% (128/445)	22.0% (11/50)	27.9% (258/925)
History of hypertension	62.6% (269/430)	65.4% (291/445)	42.0% (21/50)	62.8% (581/925)
History of diabetes	19.8% (85/430)	26.5% (118/445)	8.0% (4/50)	22.4% (207/925)
History of stroke/TIA/thromboembolism	9.1% (39/430)	7.9% (35/445)	4.0% (2/50)	8.2% (76/925)
Arrhythmia history				
Atrial fibrillation	99.3% (427/430)	42.2% (188/445)	16.0% (8/50)	67.4% (623/925)
Paroxysmal	92.5% (395/427)	78.7% (148/188)	87.5% (7/8)	88.3% (550/623)
Persistent	8.0% (34/427)	20.2% (38/188)	0.0% (0/8)	11.6% (72/623)
Longstanding persistent	0.7% (3/427)	1.6% (3/188)	12.5% (1/8)	1.1% (7/623)
Any previous treatment	90.2% (385/427)	91.5% (172/188)	100.0% (8/8)	90.7% (565/623)
Medication	91.4% (352/385)	96.5% (166/172)	100.0% (8/8)	93.1% (526/565)
Cardioversion	34.3% (132/385)	30.2% (52/172)	25.0% (2/8)	32.9% (186/565)
Ablation	26.5% (102/385)	12.8% (22/172)	50.0% (4/8)	22.7% (128/565)
Other	1.0% (4/385)	1.2% (2/172)	0.0% (0/8)	1.1% (6/565)
Atrial flutter	30.7% (132/430)	96.4% (429/445)	12.0% (6/50)	61.3% (567/925)
Typical	56.1% (74/132)	91.6% (393/429)	83.3% (5/6)	83.2% (472/567)
Atypical	13.6% (18/132)	4.2% (18/429)	0.0% (0/6)	6.3% (36/567)
Unknown	30.3% (40/132)	4.2% (18/429)	16.7% (1/6)	10.4% (59/567)
Any previous treatment	81.8% (108/132)	79.0% (339/429)	83.3% (5/6)	79.7% (452/567)
Medication	76.9% (83/108)	90.9% (308/339)	80.0% (4/5)	87.4% (395/452)
Cardioversion	25.9% (28/108)	26.0% (88/339)	20.0% (1/5)	25.9% (117/452)
Ablation	35.2% (38/108)	11.8% (40/339)	20.0% (1/5)	17.5% (79/452)
Other	0.9% (1/108)	0.0% (0/339)	0.0% (0/5)	0.2% (1/452)

### EnSite NavX patch placement

The standard patch kit (EnSite Precision NavX™ SE Patch Kit, Model EN0020-P) was used for almost all subjects in the analysis population (99.7%, 922/925) with older models used for the remaining three subjects. Patch size was reported to be appropriate for all but one subject. Standard patch placements were used in > 99% of subjects for all but the neck (91.5%, 845/924) and left leg (83.7%, 773/924) patches. Placement of the system reference surface electrode varied, with most placed on the lower back (74.5%, 689/925), followed by the upper back (15.0%, 139/925), abdomen (7.1%, 66/925), or other location (3.4%, 31/925). Positional reference sensors needed to be manually removed or repositioned in 2.8% (26/924) of subjects.

### Primary mapping catheter

For PI-AF subjects, Advisor™ FL Sensor Enabled™ (33.2%, 128/385), Advisor™ HD Grid 23.4%, 90/385), and Reflexion Spiral™ (18.2%, 70/385) were used most often as the primary mapping catheter. In contrast, an ablation catheter (53.9%, 174/323) or other linear catheter (34.4%, 111/323) was used most often for mapping in PI-AFL subjects. In PI-O subjects, Advisor™ HD Grid (23.9%, 11/46) and Tacticath™ (Quartz or SE, 21.7%, 10/46) were used most frequently as the primary mapping catheter.

### Mapping characteristics

**Fig. 2:**
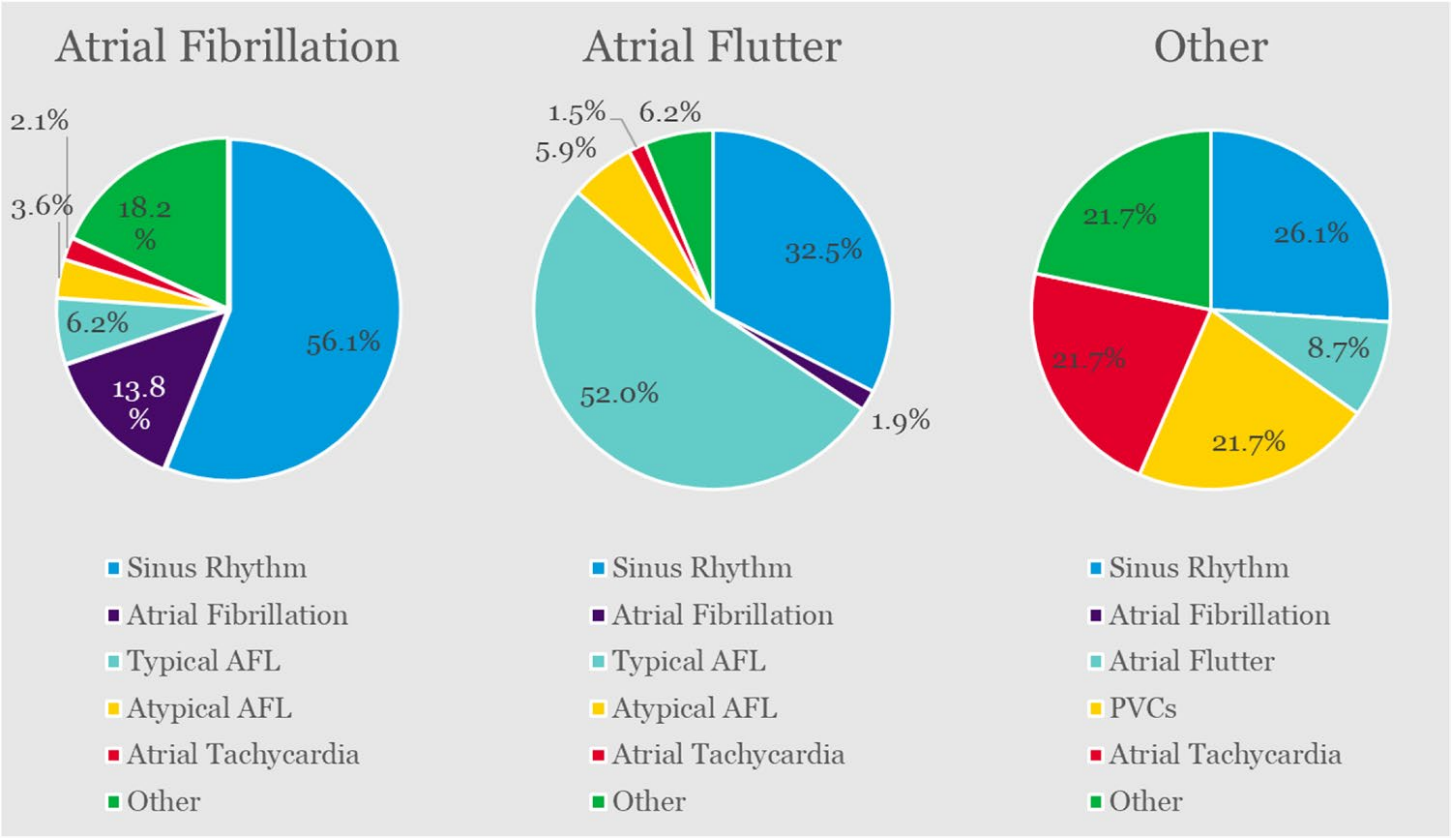
Primary cardiac rhythm during mapping

Mapping was required in 81.5% (754/925) of subjects, with OneMap (simultaneous model and electroanatomic map creation) used in 96.3% (726/754) of subjects. AutoMap was used to create the initial map in 81.8% (315/385) of PI-AF subjects, 55.7% (180/323) of PI-AFL subjects, and 28.3% (13/46) of PI-O subjects, and a combination of AutoMap and manual mapping was also used in 10.6% (41/385), 24.1% (78/323), and 32.6% (15/46) of subjects in each cohort, respectively. Sinus rhythm (56.1%, 216/385), AFL (58.2%, 188/323), and sinus rhythm (26.1%, 12/46) were the most frequent cardiac rhythms during initial map creation in each cohort, respectively (Fig. 2). Among all subjects in the analysis population, local activation time (52.0%, 392/754) and peak-to-peak voltage (46.3%, 349/754) were the predominant initial map type configurations. The low-voltage ID feature was often used in the initial maps (74.5%, 562/754).

Median time to create and edit initial map was 8.6 (IQR 4.7–15.0) and 1.0 (IQR 1.0–2.0) minutes, respectively. Only 335/925 (36.2%) required editing and 66.0% (221/335) of those required editing of fewer than 10 points. Median number of mapping points collected and used was 1752.5 and 811.0, respectively. Table 2 summarizes mapping time and point collection characteristics for the initial maps created, stratified by primary indication cohort.

**Table 2:** Mapping points and time – initial maps only

	AF	AFL	Other	All
	*N* = 430	*N* = 445	*N* = 50	*N* = 925
Mapping points used				
Median (Q1, Q3)	1570.0 (1021.0, 2214.0)	193.0 (48.0, 499.5)	257.0 (40.0, 689.0)	811.0 (173.0, 1696.0)
Mapping points collected				
Median (Q1, Q3)	4155.5 (2059.0, 7735.0)	381.0 (82.0, 1201.0)	449.0 (64.0, 2507.0)	1752.5 (307.0, 5280.0)
Number of mapping points edited				
Not applicable				
Less than 10 points edited	57.4% (93/162)	76.2% (112/147)	61.5% (16/26)	66.0% (221/335)
10–50 points edited	30.2% (49/162)	21.1% (31/147)	34.6% (9/26)	26.6% (89/335)
51–100 points edited	10.5% (17/162)	2.0% (3/147)	3.8% (1/26)	6.3% (21/335)
More than 100 points edited	1.9% (3/162)	0.7% (1/147)	0.0% (0/26)	1.2% (4/335)
Time to edit initial map (min)				
Median (Q1, Q3)	1.0 (1.0, 2.0)	1.0 (0.5, 2.0)	1.0 (1.0, 3.0)	1.0 (1.0, 2.0)
Time to create initial map (min)				
Median (Q1, Q3)	10.0 (7.0, 15.0)	5.0 (3.0, 11.0)	12.0 (5.0, 35.0)	8.6 (4.7, 15.0)

In addition to 754 initial maps, 579 additional maps were created for a total of 1333 maps. Median time to create and edit any map was 6.0 (IQR 3.0–12.0) and 1.0 (IQR 0.5–2.0) minutes, respectively. Editing of the map was not required or not applicable for most maps created (54.4%, 722/1327), and 30.2% (401/1327) required editing of fewer than 10 points. Median number of mapping points collected and used was 933.0 and 415.0, respectively. Average mapping efficiency for maps created with AutoMap or TurboMap was 164.9 ± 365.7 used points per minute (*n* = 930 maps), which was significantly greater compared to 21.8 ± 30.3 used points per minute (*n* = 374 maps) for manual mapping alone (*p* < 0.001). Table 3 summarizes mapping time and point collection characteristics for all maps created (both initial and additional), stratified by primary indication cohort. Furthermore, Supplemental Table 3 describes the differences in points taken, mapping time, fluoroscopy time, and procedure time by each mapping catheter used stratified by PI-AF and PI-AFL.

**Table 3:** Mapping points and time for all maps created

	AF	AFL	Other	All
	*N* = 430	*N* = 445	*N* = 50	*N* = 925
Mapping points used				
Median (Q1, Q3)	1012.0 (216.0, 1887.0)	148.0 (40.5, 402.0)	132.0 (24.0, 672.0)	415.0 (58.0, 1373.0)
Mapping points collected				
Median (Q1, Q3)	2413.0 (344.0, 6001.0)	303.0 (65.0, 1012.0)	239.5 (33.0, 2821.0)	933.0 (102.0, 3892.0)
Mapping points edited				
Not applicable	56.0% (423/755)	54.6% (263/482)	40.0% (36/90)	54.4% (722/1327)
Less than 10 points edited	26.2% (198/755)	34.9% (168/482)	38.9% (35/90)	30.2% (401/1327)
10–50 points edited	13.5% (102/755)	9.5% (46/482)	20.0% (18/90)	12.5% (166/1327)
51–100 points edited	3.4% (26/755)	0.6% (3/482)	1.1% (1/90)	2.3% (30/1327)
More than 100 points edited	0.8% (6/755)	0.4% (2/482)	0.0% (0/90)	0.6% (8/1327)
Time to edit map				
Median (Q1, Q3)	1.0 (0.8, 2.0)	1.0 (0.5, 2.0)	1.0 (1.0, 3.0)	1.0 (0.5, 2.0)
Time to create map				
Median (Q1, Q3)	7.0 (3.0, 12.0)	5.0 (2.0, 10.0)	8.5 (3.0, 20.0)	6.0 (3.0, 12.0)

Redo AF ablation consisted of 40/430 (23.7%) of the PI-AF cohort and redo AFL ablation consisted of 40/445 (9.8%) of the PI-AFL cohort as shown in Supplemental Table 4. As compared to de novo procedures, time creating initial map was significantly longer and RF time was shorter in redo AF procedures. As compared to de novo AFL procedures, those undergoing redo ablations had a longer procedure time, although no difference in time creating initial map, RF time, or fluoroscopy time.

### System stability

The EnSite Precision™ System was stable throughout 79.8% (738/925) of procedures. Baseline patient and procedural characteristics, including fluoroscopy and procedure times were significantly longer when the mapping system was not stable throughout the procedure (Supplemental Table 5). As displayed in Table 4, the most common factors affecting system stability were respiratory change (43.9%, 82/187), subject movement (38.0%, 71/187), and coronary sinus (CS) positional reference dislodgement (17.1%, 32/187). There were 49 “Other, specify” responses, the most frequent being Blood Pressure or Hemodynamic change (17/49) and Unknown cause (8/49). General anesthesia was used in most cases (63.0%, 583/925) with jet ventilation utilized in 10.1% (59/583). As compared to general anesthesia, those undergoing conscious sedation were more likely to have respiratory changes (4.3% vs 15.8%, *p* < 0.001), patient movement (12.7% vs 4.5%, *p* < 0.001), and no difference in CS positional reference dislodgement (3.3% vs 5.8%, *p* = 0.15). Between JET ventilation and non-JET ventilation, there were no differences in respiratory changes (3.4% vs 4.4%, *p* = 1.0), patient movement (3.4% vs 4.6%, *p* = 1.0), or CS positional reference dislodgement (0% vs 3.6%, *p* = 0.24).

**Table 4:** Ensite Precision system stability

	AF	AFL	Other	All
	*N* = 430	*N* = 445	*N* = 50	*N* = 925
Was the EnSite Precision system stable throughout the procedure?				
Yes	78.8% (339/430)	80.9% (360/445)	78.0% (39/50)	79.8% (738/925)
If no, factors affect system stability				
Respiratory change	27.5% (25/91)	58.8% (50/85)	63.6% (7/11)	43.9% (82/187)
Subject movement	27.5% (25/91)	47.1% (40/85)	54.5% (6/11)	38.0% (71/187)
CS Positional Reference dislodgement	23.1% (21/91)	10.6% (9/85)	18.2% (2/11)	17.1% (32/187)
Cardioversion	11.0% (10/91)	4.7% (4/85)	0.0% (0/11)	7.5% (14/187)
Heart rhythm change	11.0% (10/91)	3.5% (3/85)	0.0% (0/11)	7.0% (13/187)
Change in fluid load	11.0% (10/91)	0.0% (0/85)	9.1% (1/11)	5.9% (11/187)
Metal distortion	3.3% (3/91)	2.4% (2/85)	9.1% (1/11)	3.2% (6/187)
Patch movement	1.1% (1/91)	0.0% (0/85)	18.2% (2/11)	1.6% (3/187)
Other	39.6% (36/91)	12.9% (11/85)	18.2% (2/11)	26.2% (49/187)
Anesthesia used				
General anesthesia	94.0% (404/430)	37.8% (168/445)	22.0% (11/50)	63.0% (583/925)
Jet ventilation used	13.1% (53/404)	3.0% (5/168)	9.1% (1/11)	10.1% (59/583)
Intraoperative sedation (low-dose propofol drip)	1.2% (5/430)	29.4% (131/445)	32.0% (16/50)	16.4% (152/925)
Conscious sedation	4.9% (21/430)	32.6% (145/445)	46.0% (23/50)	20.4% (189/925)
No sedation nor anesthesia used	0.0% (0/430)	0.2% (1/445)	0.0% (0/50)	0.1% (1/925)

### AutoMark settings

**Fig. 3:**
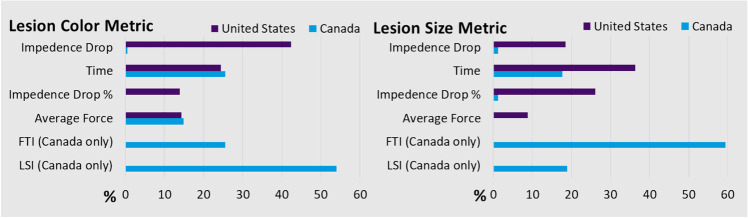
Lesion color and size metrics stratified by site of enrollment (USA and Canada)

AutoMark usage data were submitted for 755 (81.6%) of subjects. The available choices for the lesion color and size metrics are different in Canada and the USA (US); therefore, these settings are summarized by country in Fig. 3. In Canada, Force Time Integral (FTI) and Lesion Index (LSI) were available metric choices, while they were not available in the USA at that time. In Canada, the most frequently used metrics for lesion color were LSI (54.0%, 87/161), Time (25.5%, 41/161), and Average Force (14.9%, 24/161); and the most frequently used metrics for lesion size were FTI (59.5%, 47/79), LSI (19.0%, 15/79), and Time (17.7%, 14/79). In the USA, the most frequently used metrics for lesion color were Impedance Drop (42.4%, 248/585), Time (24.4%, 143/585), Average Force (14.4%, 84/585), and Impedance Drop Percent (14.0%, 82/585); and the most frequently used metrics for lesion size were Time (36.4%, 139/382), Impedance Drop Percent (26.2%, 100/382), and Impedance Drop (18.6%, 71/382).

### Procedural characteristics

Table 5 summarizes acute procedural success rates, endpoints achieved, and additional procedural characteristics. Acute success was reached based on the pre-defined endpoints for the procedure in 97.4% (901/925) of cases. Median overall procedure time (first catheter in to last catheter out) was 101.0 (IQR 59.0–152.0) minutes for all subjects, with median times within each cohort of 140.5, 59.0, and 127.0 min for PI-AFL, PI-AF, and PI-O, respectively. Among subjects with AutoMark data, an average RF power greater than 40 W was used in 10.3% (71/690) of subjects and ≥ 50 W in 17/690 (2.4%), suggesting high power short duration ablation technique may have been used in these subjects. Fluoroscopy was used for most but not all subjects (87.7%, 811/925), with the lowest proportion of fluoroscopy use in the PI-AFL cohort (83.8%, 373/445). Among procedures where fluoroscopy was used, median fluoroscopy time was 11.0 (IQR 6.0–18.0) minutes. Gaps in lesions requiring touch-up ablation were identified in 42.9% (395/921) of subjects. Median number of gaps identified was 2.0 (IQR 1.0–4.0) and AutoMark assisted in identifying the gaps in a majority of subjects with identified gaps (70.6%, 218/309).

**Table 5:** Acute procedural success, gaps requiring touch-up ablation, and overall procedure and fluoroscopy times

	AF	AFL	Other	All
	*N* = 430	*N* = 445	*N* = 50	*N* = 925
Was acute success reached based on the pre-defined endpoints for this procedure?				
Yes	98.1% (422/430)	97.5% (434/445)	90.0% (45/50)	97.4% (901/925)
If yes, which endpoint(s) were achieved				
Abolition of all clinical ventricular ectopies and unstable ventricular arrhythmias	0.0% (0/422)	0.5% (2/434)	20.0% (9/45)	1.2% (11/901)
Bidirectional block across CTI	36.0% (152/422)	95.9% (416/434)	8.9% (4/45)	63.5% (572/901)
Pulmonary vein electrical isolation	87.4% (369/422)	2.8% (12/434)	6.7% (3/45)	42.6% (384/901)
Pulmonary vein capture with exit block	60.2% (254/422)	2.3% (10/434)	6.7% (3/45)	29.6% (267/901)
Termination of tachycardia during RF energy application	8.1% (34/422)	12.7% (55/434)	24.4% (11/45)	11.1% (100/901)
Elimination and noninducibility of tachycardia following ablation	10.0% (42/422)	6.5% (28/434)	46.7% (21/45)	10.1% (91/901)
Block across mitral isthmus	3.6% (15/422)	0.5% (2/434)	0.0% (0/45)	1.9% (17/901)
Other	14.0% (59/422)	8.3% (36/434)	24.4% (11/45)	11.8% (106/901)
Subjects with gaps in lesions identified that require touch-up ablation	46.5% (199/428)	43.1% (191/443)	10.0% (5/50)	42.9% (395/921)
Number of gaps				
Median (Q1, Q3)	3.0 (2.0, 5.0)	2.0 (1.0, 3.0)	1.0 (1.0, 3.0)	2.0 (1.0, 4.0)
Subjects with AutoMark assisted in identifying gaps	75.3% (113/150)	66.0% (103/156)	66.7% (2/3)	70.6% (218/309)
Overall procedure time				
Median (Q1, Q3)	140.5 (104.0, 190.0)	59.0 (34.0, 91.0)	127.0 (98.0, 179.0)	101.0 (59.0, 152.0)
Fluoroscopy used				
Yes,	91.6% (394/430)	83.8% (373/445)	88.0% (44/50)	87.7% (811/925)
If yes, fluoroscopy time				
Median (Q1, Q3)	15.0 (10.0, 20.0)	7.0 (3.0, 13.0)	9.5 (5.0, 18.0)	11.0 (6.0, 18.0)

## Discussion

In this real-world, multi-center study including 925 patients undergoing mapping and ablation using the EnSite Precision™ cardiac mapping system for a variety of arrythmias, we demonstrated several key observations: (1) there was high procedural stability in nearly 80% of patients; (2) the system allows for high point density collection and short mapping times with the aid of AutoMap and TurboMap; (3) maps required editing in slightly over a third of patients with two-thirds requiring editing of fewer than 10 points; and (4) acute procedural success was high for all procedures.

The automated 3D mapping system, EnSite Precision™, uses a hybrid magnetic and impedance-based catheter technology to accurately locate ablation catheters and create electroanatomic maps. A high-frequency (8 kHz) signal is sent through the three pairs of surface electrodes to interact with the sensor-enabled catheters to create a voltage gradient in three axes of space. A catheter is used as reference (typically in the CS) and after analysis of the voltages and impedance gradient, the localizations of catheters are determined within the cardiac chamber. To increase accuracy to less than 1 mm, a weak magnetic field is generated by a field frame attached under the table that is employed to enhance the impedance-based tracking [4, 5]. Taken together, the use of the hybrid system allows for an accurate and stable mapping system, as demonstrated by stability in nearly 80% of the current study. Subject movement and respiratory changes were the most common causes of system instability, followed by CS catheter dislodgement. To ensure stability, placement of stable reference via the CS catheter should be confirmed. Sedation type was shown to influence stability by preventing patient movement and respiratory changes. Moderate sedation was more common in the unstable cohort (23.5% vs 19.6%) as compared to general anesthesia and accounted for more respiratory changes and patient movement, suggesting general anesthesia may be favored to maintain stability. Although jet ventilation has demonstrated improved stability in prior studies, our comparison was limited by small number of patients in the jet ventilation arm [6]. Efforts to understand strategies beyond sedation type to improve stability are warranted.

The Ensite Precision system is an open-platform system that permits catheters from different manufacturers to be used to generate a map. For AF, the most used mapping catheter was the Advisor FL Sensor Enabled (33.2%), followed by the Advisor HD Grid (23.4%), while a more variable selection was observed for AFL with “Other” encompassing 25.4% of the catheters use, likely mostly catheters from other manufacturers. Further advancements in mapping technology allow for collection of multiple points simultaneously to rapidly build EAMs. The AutoMap feature allows for rapid signal discrimination without the need for operator discretion allowing for nearly continuous movement of the catheter for EAM creation. The use of the AutoMap feature has been shown to result in significantly faster mapping times with higher point density than manual, point-by-point mapping [3]. In this study reflecting real-world practice, over 80% of the maps for atrial fibrillation indication and approximately 56% of those for atrial flutter were created using the Automap feature requiring a median of 10 min and 5 min, respectively, of mapping time with high point density. In addition, only roughly a third of patients required map editing with the majority requiring editing of fewer than 10 points in a median time of 1 min, allowing for a time-efficient process to create accurate EAMs.

Lastly, there was high acute procedural success across the indications, including approximately 98% for AF and AFL. There currently exist no randomized trials comparing mapping systems with respect to success rates of specific arrhythmias, although a few prior observational studies have described acute procedural success with other mapping systems in various arrhythmias. In 1,070 consecutive patients referred for RF catheter ablation for all arrhythmias, Romero et al. observed no difference in acute procedural success between CARTO (Biosense, Diamond Bar, CA, USA) (88.2%) and Ensite NavX (91.1%) [7]. In a separate single-center study of 70 patients undergoing focal atrial tachycardia ablation comparing the acute procedural outcomes between CARTO (*n* = 22) and Rhythmia (Boston Scientific) (*n* = 48) mapping systems, Kellnar et al. observed significantly higher success rates in the Rhythmia cohort (89.6% vs 68.2%, *p* = 0.03) [8]. Lastly, in another study comparing CARTO and Rhythmia mapping systems in 74 patients undergoing AF ablation, there was no difference in acute procedural success as PVI was achieved in all patients, although Rhythmia resulted in shorter mapping times [9]. We expand on prior studies by describing the first systematic characterization of the Ensite Precision mapping system in a large multicenter study in patients undergoing various arrhythmias reflecting real-world practice. Although no comparison was used to adequately determine effectiveness of the mapping system in achieving acute procedural success, a few additional features are worth highlighting that may contribute to positive outcomes. For instance, unique features of the mapping system include customizable lesion color and size metrics. In Canada, the most used metrics include LSI for lesion color and FTI for lesion size, both of which rely on contact force, while the USA commonly used impedance drop percent for lesion color and time for lesion size. As there is no single parameter currently that best identifies durable lesion formation, emerging data has supported the use of contact force-sensing catheters, LSI, FTI, and impedance drop percent for determining lesion efficacy and predicting gaps [10–13]. In our study, roughly 45% of patients in both AF and AFL cohorts required touch-up ablation of identified gaps, with a median of 3 and 2 gaps, respectively. Of note, those with gaps requiring touch-up do not imply failure of first-pass isolation, which was not captured in the current study. Gaps could include those identified after first-pass failure to isolate the PVs, gaps identified in PVs with demonstrated isolation after first pass that later reconnected during the procedure, or visual gaps that were further ablated by the operator irrespective of successful isolation. The automated lesion documentation tool, AutoMark, as opposed to manual marking, was used in nearly 71% of cases to identify visual gaps. While automated features increase procedural efficiency and shorten procedural times, further studies are needed to determine whether automated marking features better localize lesions as compared to manual marking and ultimately result in decreased adverse complications and improve long-term success.

### Limitations

The present study must be interpreted in the context of several limitations inherent to its design. First, as an observational study including only one mapping system, comparisons to other systems cannot be made. Rather, these results validate the efficacy of the EnSite Precision™ mapping system. Second, the cohort consisted of ablation for various types of arrhythmias producing significant heterogeneity in some findings. We believe this accurately reflects clinical practice and allows for generalizability of our observations. Still, efforts were made to stratify according to arrhythmia type, although other unmeasured factors likely influence the outcomes studied, such as use of intracardiac ultrasound, provider experience, and need for additional ablation lesions. Third, adverse outcomes during the procedure were not recorded. Fourth, utilization of general anesthesia and jet ventilation continues to increase, and the present study may underestimate system stability in practice today. Finally, these data represent outcomes from a single mapping system that has undergone engineering and user improvements. The newer generation system, EnSite™ X EP System, will aim to broaden the range of mapping capabilities with further improvements on procedural efficiency and success.

## Conclusion

This real-world study demonstrates that use of the open-platform EnSite Precision™ mapping system results in high procedural stability, short mapping times, high point density with the use of Auto/Turbo map requiring infrequent editing, low fluoroscopy time, and high prevalence of acute procedural success.

*Three (3) subjects were enrolled in the Primary Indication Atrial Fibrillation cohort without the study site confirming history of atrial fibrillation. Of these, 1 subject received ablation for atrial fibrillation and atrial flutter, 1 subject received ablation for atrial fibrillation, and 1 subject received ablation for atrial flutter.

## Supplementary Information

Below is the link to the electronic supplementary material.

Supplementary file1 (DOCX 55 kb)

## Abbreviations

EAM: Electroanatomic mapping
EDC: Electronic data capture
AVNRT: Atrioventricular nodal reentrant tachycardia
AVRT: Atrioventricular reentrant tachycardia
IFU: Instructions for Use
AF: Atrial fibrillation
AFL: Atrial flutter
SVT: Supraventricular tachycardia
PVC: Premature ventricular contractions
RF: Radiofrequency
CS: Coronary sinus
FTI: Force Time Integral
LSI: Lesion Index

## References

[1] Shpun S, Gepstein L, Hayam G, Ben-Haim SA (1997). Guidance of radiofrequency endocardial ablation with real-time three-dimensional magnetic navigation system. Circulation.

[2] Koruth JS, Heist EK, Danik S, Barrett CD, Kabra R, Blendea D, Ruskin J, Mansour M (2011). Accuracy of left atrial anatomical maps acquired with a multielectrode catheter during catheter ablation for atrial fibrillation. J Interv Card Electrophysiol.

[3] Ptaszek LM, Moon B, Rozen G, Mahapatra S, Mansour M (2018). Novel automated point collection software facilitates rapid, high-density electroanatomical mapping with multiple catheter types. J Cardiovasc Electrophysiol.

[4] Borlich M, Sommer P (2019). Cardiac mapping systems: Rhythmia, Topera, EnSite Precision, and CARTO. Card Electrophysiol Clin.

[5] Bourier F, Gianni C, Dare M, Deisenhofer I, Hessling G, Reents T, Mohanty S, Trivedi C, Natale A, Al-Ahmad A (2017). Fiberoptic contact-force sensing electrophysiological catheters: how precise is the technology?. J Cardiovasc Electrophysiol.

[6] Osorio J, Varley A, Kreidieh O, Godfrey B, Schrappe G, Rajendra A, Silverstein J, Romero J, Rodriguez D, Morales G et al (2022) High-frequency, low-tidal-volume mechanical ventilation safely improves catheter stability and procedural efficiency during radiofrequency ablation of atrial fibrillation. Circ Arrhythm Electrophysiol CIRCEP121010722. 10.1161/CIRCEP.121.010722 10.1161/CIRCEP.121.010722 35333095

[7] Romero J, Lupercio F, Goodman-Meza D, Ruiz JC, Briceno DF, Fisher JD, Gross J, Ferrick K, Kim S, Di Biase L (2016). Electroanatomic mapping systems (CARTO/EnSite NavX) vs. conventional mapping for ablation procedures in a training program. J Interv Card Electrophysiol..

[8] Kellnar A, Fichtner S, Mehr M, Czermak T, Sinner MF, Lackermair K, Estner HL (2022). Single-center experience of ultra-high-density mapping guided catheter ablation of focal atrial tachycardia. Clin Cardiol.

[9] Rottner L, Metzner A, Ouyang F, Heeger C, Hayashi K, Fink T, Lemes C, Mathew S, Maurer T, Reißmann B (2017). Direct comparison of point-by-point and rapid ultra-high-resolution electroanatomical mapping in patients scheduled for ablation of atrial fibrillation. J Cardiovasc Electrophysiol.

[10] Kanamori N, Kato T, Sakagami S, Saeki T, Kato C, Kawai K, Chikata A, Takashima SI, Murai H, Usui S (2018). Optimal lesion size index to prevent conduction gap during pulmonary vein isolation. J Cardiovasc Electrophysiol.

[11] Neuzil P, Reddy VY, Kautzner J, Petru J, Wichterle D, Shah D, Lambert H, Yulzari A, Wissner E, Kuck KH (2013). Electrical reconnection after pulmonary vein isolation is contingent on contact force during initial treatment: results from the EFFICAS I study. Circ Arrhythm Electrophysiol.

[12] Garrott K, Laughner J, Gutbrod S, Sugrue A, Shuros A, Sulkin M, Yasin O, Bush J, Pottinger N, Meyers J (2020). Combined local impedance and contact force for radiofrequency ablation assessment. Heart Rhythm.

[13] Reddy VY, Dukkipati SR, Neuzil P, Natale A, Albenque JP, Kautzner J, Shah D, Michaud G, Wharton M, Harari D (2015). Randomized, controlled trial of the safety and effectiveness of a contact force-sensing irrigated catheter for ablation of paroxysmal atrial fibrillation: results of the TactiCath Contact Force Ablation Catheter Study for Atrial Fibrillation (TOCCASTAR) study. Circulation.

