# Supplementary material for: Performance and acute procedural outcomes of the EnSite Precision™ cardiac mapping system for electrophysiology mapping and ablation procedures: results from the EnSite Precision™ observational study
Source: J Interv Card Electrophysiol. 2022 May 10;65(1):141–51. doi: 10.1007/s10840-022-01239-4 (PMC9550718; doi:10.1007/s10840-022-01239-4)
Supplement: Supplementary file 1 — Supplementary file1 (DOCX 55 kb) [file 10840_2022_1239_MOESM1_ESM.docx]

**Supplemental Table 1:** Inclusion/Exclusion Criteria

| **Inclusion Criteria**   - Indicated for a cardiac EP mapping and RF ablation procedure using a 3D mapping system per IFU - Over 18 years of age - Able to provide informed consent for study participation and willing and able to comply with the protocol described evaluations and follow up schedule   **Exclusion Criteria**   - Patients who are only presenting with:   - Atrioventricular Nodal Reentrant Tachycardia (AVNRT)   - Atrioventricular Reentrant Tachycardia (AVRT) - Planned cryoablation procedure - Implanted with a neurostimulator - Contraindication to anticoagulation - Known presence of cardiac thrombus - Recent (<3 months) myocardial infarction or unstable angina or coronary artery by-pass - Currently enrolled in a clinical study/investigation evaluating another device or drug that would confound the results of this study - Pregnant or nursing - Individuals whose willingness to volunteer in a study, in the judgement of investigator or public authorities, could be unduly influenced by lack of or loss of the autonomy due to immaturity, or mental disability, or adverse personal circumstances, or hierarchical influence |
| --- |

**Supplemental Table 2:** Lesions performed in the patients undergoing atrial fibrillation ablation.

|  | **AF Subjects (n=430)** |
| --- | --- |
|  |  |
| **Pulmonary Vein Isolation** | 88.4% (380/430) |
| Right | 4.5% (17/380) |
| Left | 3.2% (12/380) |
| Right/Left | 92.4% (351/380) |
| **Right Sided Flutter** | 34.7% (149/430) |
| CTI-dependent | 97.3% (145/149) |
| Non CTI-dependent | 2.7% (4/149) |
| **Rotor/Foci** | 2.3% (10/430) |
| **Complex Fractionated Atrial Electrogram** | 4.0% (17/430) |
| **Lines** | 8.8% (38/430) |
| **Substrate Based Ablation** | 4.4% (19/430) |
| **Slow Pathway Ablation** | 1.4% (6/430) |
| **Other** | 7.0% (30/430) |

**Supplemental Table 3:** Procedure characteristics by mapping catheter stratified by atrial fibrillation and atrial flutter.

|  | **AF  (n=430)** | **AFL  (n=445)** |
| --- | --- | --- |
|  |  |  |
| **Mapping Points Used** |  |  |
| Advisor FL | 1359.5 ± 664.1 (126) | 849.0 ± 542.2 (12) |
| Advisor HD | 2303.2 ± 889.6 (90) | 1850.1 ± 1756.2 (21) |
| Reflexion Spiral | 1937.4 ± 698.6 (70) | 269.0 ± NA (1) |
| TactiCath | 924.1 ± 841.2 (31) | 206.2 ± 254.3 (63) |
| Inquiry AFocus II | 2433.3 ± 1284.5 (12) | 2676.0 ± 470.9 (2) |
| Inquiry AFocus II DL | 1352.2 ± 1268.7 (6) | 5394.0 ± NA (1) |
| Reflexion Spiral HD | 2135.9 ± 1031.4 (8) | NA |
| Livewire EP | 417.0 ± NA (1) | 271.0 ± 318.4 (53) |
| FlexAbility/Flex SE | 1033.0 ± 1428.4 (2) | 142.5 ± 166.6 (85) |
| Other | 1126.2 ± 750.4 (35) | 442.2 ± 352.4 (82) |
| **Mapping Points Collected** |  |  |
| Advisor FL | 3049.3 ± 2285.4 (127) | 1603.9 ± 1477.1 (12) |
| Advisor HD | 11715.4 ± 13175.4 (90) | 13316.1 ± 17823.1 (21) |
| Reflexion Spiral | 5963.0 ± 3435.0 (70) | 449.0 ± NA (1) |
| TactiCath | 2347.4 ± 2833.4 (31) | 762.4 ± 1497.4 (63) |
| Inquiry AFocus II | 13007.8 ± 5747.9 (12) | 13428.0 ± 10772.1 (2) |
| Inquiry AFocus II DL | 2790.5 ± 2243.6 (6) | 11559.0 ± NA (1) |
| Reflexion Spiral HD | 7163.0 ± 5828.8 (8) | NA |
| Livewire EP | 210.0 ± NA (1) | 888.5 ± 1885.2 (53) |
| FlexAbility/Flex SE | 3275.0 ± 4593.4 (2) | 349.1 ± 718.9 (85) |
| Other | 2728.0 ± 2256.2 (35) | 1104.7 ± 1107.6 (81) |
| **Time to Edit Initial Map** |  |  |
| Advisor FL | 2.1 ± 1.6 (55) | 1.4 ± 1.5 (9) |
| Advisor HD | 1.7 ± 1.7 (33) | 2.6 ± 2.6 (15) |
| Reflexion Spiral | 2.5 ± 5.5 (31) | NA |
| TactiCath | 1.3 ± 1.1 (7) | 1.4 ± 1.5 (16) |
| Inquiry AFocus II | 1.3 ± 1.8 (7) | 1.5 ± 0.7 (2) |
| Inquiry AFocus II DL | 1.0 ± NA (1) | NA |
| Reflexion Spiral HD | 1.0 ± 0.0 (2) | NA |
| Livewire EP | NA | 1.5 ± 1.2 (29) |
| FlexAbility/Flex SE | 1.5 ± 0.7 (2) | 1.5 ± 2.1 (24) |
| Other | 4.8 ± 11.9 (17) | 1.8 ± 2.1 (43) |
| **Time to Create Initial Map** |  |  |
| Advisor FL | 11.2 ± 6.0 (128) | 7.5 ± 6.2 (12) |
| Advisor HD | 13.8 ± 8.0 (90) | 12.7 ± 9.4 (21) |
| Reflexion Spiral | 10.4 ± 11.8 (70) | 5.0 ± NA (1) |
| TactiCath | 18.8 ± 15.6 (31) | 12.1 ± 14.1 (62) |
| Inquiry AFocus II | 12.9 ± 7.6 (12) | 18.0 ± 15.6 (2) |
| Inquiry AFocus II DL | 4.3 ± 2.4 (6) | 15.0 ± NA (1) |
| Reflexion Spiral HD | 17.4 ± 6.6 (8) | NA |
| Livewire EP | 9.0 ± NA (1) | 4.2 ± 5.3 (52) |
| FlexAbility/Flex SE | 13.0 ± 4.2 (2) | 8.0 ± 10.8 (86) |
| Other | 11.7 ± 12.3 (35) | 7.5 ± 5.8 (82) |
| **Fluoro Time** |  |  |
| Advisor FL | 14.7 ± 8.0 (123) | 19.0 ± 11.3 (2) |
| Advisor HD | 14.5 ± 8.7 (72) | 26.7 ± 45.7 (18) |
| Reflexion Spiral | 15.0 ± 6.8 (70) | 3.0 ± NA (1) |
| TactiCath | 19.5 ± 9.5 (31) | 7.0 ± 6.3 (62) |
| Inquiry AFocus II | 10.8 ± 7.0 (12) | 11.5 ± 9.2 (2) |
| Inquiry AFocus II DL | 23.3 ± 6.5 (6) | 19.0 ± NA (1) |
| Reflexion Spiral HD | 14.6 ± 4.4 (8) | NA |
| Livewire EP | 6.0 ± NA (1) | 9.9 ± 5.7 (50) |
| FlexAbility/Flex SE | 14.0 ± 1.4 (2) | 9.6 ± 10.2 (73) |
| Other | 23.8 ± 12.1 (34) | 10.7 ± 10.1 (78) |
| **Procedure Time** |  |  |
| Advisor FL | 155.7 ± 86.4 (128) | 86.7 ± 72.6 (12) |
| Advisor HD | 170.9 ± 61.4 (90) | 132.2 ± 78.1 (21) |
| Reflexion Spiral | 123.5 ± 67.3 (70) | 32.0 ± NA (1) |
| TactiCath | 200.0 ± 61.4 (31) | 90.0 ± 47.9 (65) |
| Inquiry AFocus II | 242.5 ± 206.7 (13) | 104.0 ± 4.2 (2) |
| Inquiry AFocus II DL | 105.5 ± 31.8 (6) | 71.0 ± NA (1) |
| Reflexion Spiral HD | 183.9 ± 32.1 (8) | NA |
| Livewire EP | 65.0 ± NA (1) | 51.0 ± 44.7 (53) |
| FlexAbility/Flex SE | 97.0 ± 49.5 (2) | 71.2 ± 44.0 (86) |
| Other | 164.2 ± 66.6 (36) | 73.5 ± 41.9 (82) |

**Supplemental Table 4.** Time creating initial map, radiofrequency ablation time, fluoroscopy time, and procedure time in de novo vs redo ablation stratified by atrial fibrillation and atrial flutter cohorts.

|  | AF Subjects | | | AFL | | |
| --- | --- | --- | --- | --- | --- | --- |
|  | De-novo  (n=328) | Redo  (n=102) | P-value | De-novo  (n=405) | Redo  (n=40) | P-value |
| Time Creating Initial Map | 11.5 ± 8.1 (288) | 15.1 ± 13.0 (95) | 0.013 | 8.2 ± 9.7 (292) | 11.4 ± 11.8 (28) | 0.15 |
| RF Time | 85.6 ± 334.8 (317) | 32.7 ± 56.8 (99) | 0.007 | 55.7 ± 221.7 (387) | 91.1 ± 266.2 (35) | 0.45 |
| FluoroscopyTime | 15.7 ± 8.7 (298) | 16.9 ± 10.0 (96) | 0.27 | 9.5 ± 9.0 (338) | 15.1 ± 34.4 (34) | 0.35 |
| Procedure Time | 156.5 ± 84.9 (328) | 153.7 ± 72.9 (102) | 0.74 | 66.3 ± 45.7 (405) | 93.7 ± 69.1 (40) | 0.018 |

**Supplemental Table 5:** Baseline and Procedure Characteristics by System Stability

|  | **Stable N=738** | **Unstable N=187** | **P-Value** |
| --- | --- | --- | --- |
|  |  |  |  |
| Age (Years)    Mean ± SD (n)    (Min, Max) | 64.4 ± 11.8 (738)  (20.0, 88.0) | 63.9 ± 10.6 (187)  (31.0, 88.0) | 0.61 |
| Gender |  |  | 0.41 |
| Female | 29.5% (218/738) | 32.6% (61/187) |  |
| Male | 70.5% (520/738) | 67.4% (126/187) |  |
| Height (in)    Mean ± SD (n)    (Min, Max) | 68.5 ± 4.0 (738)  (55.0, 78.0) | 68.6 ± 4.1 (187)  (59.0, 81.1) | 0.92 |
| Weight (lb)    Mean ± SD (n)    (Min, Max) | 206.6 ± 53.6 (737)  (105.6, 657.0) | 208.9 ± 47.8 (187)  (57.3, 363.8) | 0.60 |
| Body Mass Index (kg/m²)    Mean ± SD (n)    (Min, Max) | 30.8 ± 7.6 (737)  (14.9, 124.1) | 31.2 ± 6.6 (187)  (9.3, 54.6) | 0.56 |
| Implanted Cardiac Device | | |  |
| None | 83.5% (616/738) | 87.2% (163/187) | 0.22 |
| LVEF (%)    Mean ± SD (n)    (Min, Max) | 54.0 ± 12.0 (570)  (5.0, 82.0) | 55.1 ± 11.4 (141)  (15.0, 76.0) | 0.31 |
| NYHA Classification |  |  | 0.03 |
| I | 3.7% (27/738) | 3.2% (6/187) |  |
| II | 6.4% (47/738) | 4.3% (8/187) |  |
| III | 2.6% (19/738) | 0.0% (0/187) |  |
| IV | 0.0% (0/738) | 0.5% (1/187) |  |
| Not Evaluated | 87.4% (645/738) | 92.0% (172/187) |  |
| **Cardiovascular Disease** | | |  |
| Coronary artery disease | 26.7% (197/738) | 18.7% (35/187) | 0.02 |
| Myocardial infarction | 8.8% (65/738) | 6.4% (12/187) | 0.29 |
| Previous CABG | 10.0% (74/738) | 2.7% (5/187) | 0.001 |
| Percutaneous coronary intervention/stent/atherectomy | 12.6% (93/738) | 8.0% (15/187) | 0.08 |
| Cardiomyopathy | 16.3% (120/738) | 14.4% (27/187) | 0.54 |
| Valvular heart disease | 28.2% (208/738) | 26.7% (50/187) | 0.69 |
| History of hypertension | 63.1% (466/738) | 61.5% (115/187) | 0.68 |
| History of diabetes | 22.5% (166/738) | 21.9% (41/187) | 0.87 |
| History of stroke/TIA/thromboembolism | 9.1% (67/738) | 4.8% (9/187) | 0.057 |
| **Arrhythmia History** |  |  |  |
| Atrial Fibrillation | 67.8% (500/738) | 65.8% (123/187) | 0.61 |
| Atrial Flutter | 62.3% (460/738) | 57.2% (107/187) | 0.20 |
| **Intraprocedural Factors** |  |  |  |
| **Anesthesia type** |  |  | 0.02 |
| Conscious Sedation | 19.6% (145/738) | 23.5% (44/187) |  |
| General Anesthesia with Jet Ventilation | 7.0% (52/738) | 3.7% (7/187) |  |
| General Anesthesia without Jet Ventilation | 58.3% (430/738) | 50.3% (94/187) |  |
| Intraoperative Sedation | 15.0% (111/738) | 21.9% (41/187) |  |
| Fluoroscopy time, minutes | 12.6 ± 12.2 (636) | 14.4 ± 9.4 (172) | 0.04 |
| Procedure time, minutes | 109.7 ± 81.0 (737) | 127.4 ± 72.5 (187) | 0.006 |
